# The Prognostic Value and Function of HOXB5 in Acute Myeloid Leukemia

**DOI:** 10.3389/fgene.2021.678368

**Published:** 2021-08-05

**Authors:** Miao Chen, Yi Qu, Pengjie Yue, Xiaojing Yan

**Affiliations:** Department of Hematology, The First Affiliated Hospital of China Medical University, Shenyang, China

**Keywords:** HOXB5, AML, prognosis, differentiation, TNF/NF-κB, methylation

## Abstract

**Background:**

Currently, cytogenetic and genetic markers are the most important for risk stratification and treatment of patients with acute myeloid leukemia (AML). Despite the identification of many prognostic factors, relatively few have made their way into clinical practice. Therefore, the identification of new AML biomarkers is useful in the prognosis and monitoring of AML and contributes to a better understanding of the molecular basis of the disease. Homeobox (HOX) genes are transcription factors that lead to cell differentiation blockade and malignant self-renewal. However, the roles of HOX genes in AML are still not fully understood and need further exploration, which may provide new strategies for the prognosis and monitoring of AML.

**Methods:**

We analyzed the RNA sequencing and clinical data from The Cancer Genome Atlas (TCGA), VIZOME, GSE13159, and GSE9476 cohorts. Analyses were performed with GraphPad 7, the R language, and several online databases. We applied quantitative polymerase chain reaction, Western Blotting, CCK8 cell proliferation assays, and flow cytometry to verify the conclusions of the bioinformatics analysis.

**Results:**

We identified HOXB5 as the only gene among the HOX family that was not only elevated in AML but also a significant prognostic marker in AML patients. HOXB5 was highly expressed in AML patients with NPM1, FLT3, or DNMT3A mutations and was expressed at the highest level in patients with NPM1-FLT3-DNMT3A triple-mutant AML. Gene Ontology analysis and gene set enrichment analysis revealed that HOXB5 showed a negative correlation with the myeloid cell differentiation signature and that the tumor necrosis factor/nuclear factor κB signaling pathway was involved in the molecular mechanism. Moreover, we performed *in silico* protein–protein interaction analysis and 450K TCGA DNA methylation data analysis and found that HOXB5 interacted with two HOX genes (HOXA7 and HOXB4) that were commonly regulated by DNA methylation levels.

**Conclusion:**

HOXB5 is associated with the malignant development of AML and may be a treatment target and biomarker for AML prognosis prediction.

## Introduction

Acute myeloid leukemia (AML) is a hematopoietic neoplasm characterized by clonal proliferation and differentiation blockade and inhibited apoptosis of malignant stem or progenitor cells (Alharbi et al., 2013; Chen et al., 2019; Nagy et al., 2019; MacPherson et al., 2020). AML is a heterogeneous disease, and its prognosis is still unsatisfactory, with an overall 5-year survival rate of less than 50% in adults and significantly lower in the elderly (Döhner et al., 2015; Miller et al., 2016; Siegel et al., 2017). Standard therapies often fail to achieve complete remission (CR), and relapse may occur after CR (Chen et al., 2019). Therefore, it is necessary to further explore the internal mechanism of AML initiation and development, which may provide effective treatment and prognosis prediction strategies. At present, cytogenetic risk is estimated according to chromosomal structural variations and is commonly used to predict the likelihood of CR and relapse, as well as overall survival (OS) (Alharbi et al., 2013; Nagy et al., 2019). Genetic classification based on genetic mutational status further refines patient stratification according to the 2017 European Leukemia Net (ELN) recommendations (Döhner et al., 2017), but clinical uncertainty remains, with an unclassifiable group of patients not having the defined chromosomal or genetic alterations. Therefore, we need to explore new dysregulated genes to more precisely classify the cytogenetic risk.

Homeobox (HOX) genes are highly conserved homeodomain-containing transcription factors important for early development, maintaining the stemness of stem cells, and hematopoiesis (Bhatlekar et al., 2018). The human HOX gene family includes 39 members, which are located in clusters on four different chromosomes (7p15, 17q21.2, 12q13, and 2q31) and divided into four families (HOXA, HOXB, HOXC, and HOXD) (Gonçalves et al., 2020). The deregulation of HOX genes in AML and the potential for these master regulators to perturb normal hematopoiesis are well established (Sauvageau et al., 1994; Pineault et al., 2002; Andreeff et al., 2008; Eklund, 2011; Nagy et al., 2019). Elevated expression of several HOX genes, such as HOXB3, HOXB4, and HOXA7-11, has been proven to be related to disease pathogenesis and poor prognosis in AML (Eklund, 2011). Although some HOX genes have been studied in detail in AML, HOX genes actually function as a network, and it is necessary to comprehensively determine the prognostic significance of HOX genes in AML and explore the underlying mechanism.

In this study, we first identified four HOX members that were significantly upregulated in AML patients and found HOXB5 to be the only significantly altered HOX gene with prognostic value. Then, we analyzed the relationship between HOXB5 and the malignant phenotypes of AML and found that HOXB5 might participate in the progression of AML. Moreover, *in silico* enrichment analysis showed that HOXB5 was negatively associated with the regulation of myeloid cell differentiation, probably through the tumor necrosis factor (TNF)/nuclear factor κB (NF-κB) pathway. HOXB5 could be a potential biomarker for AML prognosis prediction and treatment targeting.

## Materials and Methods

### Data Collection

Transcriptome sequencing data of 542 AML patients, 206 MDS patients, and 74 healthy volunteers were collected from GSE13159^1^. Transcriptome sequencing data of 26 AML patients and 10 healthy volunteers were obtained from GSE9476^2^. One hundred seventy-nine AML patients’ clinical information and transcriptome sequencing data of The Cancer Genome Atlas (TCGA) were downloaded from https://xenabrowser.net. The AML mutation data (MAF file), copy number variation (CNV) data, and DNA methylation data were also downloaded from https://xenabrowser.net. Clinical information along with transcriptome sequencing data of VIZOME (451 patients) were downloaded from https://www.vizome.org/ and https://www.cbiopportal.org/. Transcriptome sequencing data of 17 AML cell lines were downloaded from https://www.portals.broadinstitute.org/ccle/data. Drug sensitivity data of 17 AML cell lines were obtained from http://www.cancerrxgene.org/.

### Bioinformatics Analysis

Limma R package was used to calculate differential expression genes between AML patients and healthy volunteers. Mutation analysis was done by Maftool R package. The Gene Ontology (GO) enrichment analysis was performed by DAVID 6.8^3^ to find possible functions of HOXB5. Gene set enrichment analysis (GSEA) was carried out to verify the AML-related functions and explore the potential signal pathway leading to malignant phenotypes between patients with high and low HOXB5 expression^4^. Heatmaps were made by R language to express information correlated with HOXB5 expression. HOXB5 protein–protein interaction was detected using the Search Tool for the Retrieval of Interacting Genes/Proteins (STRING) and GeneMANIA datasets. STRING and GeneMANIA are both frequently used datasets, which can provide protein–protein interaction information (Warde-Farley et al., 2010; Szklarczyk et al., 2019).

### Cell Lines and Cell Culture

The human AML cell lines THP1, KASUMI-1, HL60, and MOLM14 were purchased from the Chinese Academy of Sciences Cell Bank (Shanghai, China). The human AML cell lines were cultured in RPMI-1640 medium containing 10% fetal bovine serum and 1% penicillin/streptomycin at 37°C with 5% CO_2_.

### Antibodies and Reagents

Antibodies used were as follows: HOXB5 antibody (Abcam, ab109375), GAPDH antibody (PTG, 60004-1), horseradish peroxidase (HRP)–conjugated Affinipure Goat Anti-Mouse immunoglobulin G (IgG) (H + L) (PTG, SA00001-1), HRP-conjugated Affinipure Goat Anti-Rabbit IgG (H + L) (PTG, SA00001-2), TNF-α antibody (PTG, 60291-1-Ig), RELA antibody (CST, 8242S), p-RELA antibody (CST, 3033S), IKBα antibody (PTG, 10268-1-AP), p-IKBα antibody (Affinity, AF2002), and CD11b antibody (BD, 550019), CD14 antibody (BD, 347493). Reagents were as follows: RIPA Lysis Buffer (Beyotime Biotechnology, P0013K), TRIzol^TM^ Reagent (Invitrogen, 15596026), PrimeScript^TM^ RT Master Mix (Perfect Real Time) (TAKARA, RR036Q), TB Green^®^ Premix Ex Taq^TM^ II (Tli RNaseH Plus) (TAKARA, RR820Q), bortezomib (MCE, HY10227), daunomycin (MCE, HY13062), cytarabine (MCE, HY13605), CCK8 (Dojindo, CK04), and PMA (Sigma, P1585).

### RNA Isolation and Reverse Transcription–Quantitative Polymerase Chain Reaction

Total RNA was isolated from AML cell lines using TRIzol reagent (Invitrogen), according to the manufacturer’s instructions. Total RNA was reverse transcribed into cDNA using Prime-Script RT Master Mix (TaKaRa). Quantitative polymerase chain reaction (qPCR) was performed using SYBR Green Master Mix (TaKaRa). The following conditions were used: one cycle of 95°C for 30 s, followed by 40 cycles of a two-step cycling program (95°C for 5 s, 60°C for 30 s). The mRNA expression of target genes was calculated by the 2 ΔΔCt method and normalized to GAPDH mRNA expression.

The primers for the HOXB5 gene were as follows: forward: 5′-AACTCCTTCTCGGGGCGTTAT-3′; reverse: 5′-CATCCCAT TGTAATTGTAGCCGT-3′.

The primers for the SPI1 gene were as follows: forward: 5′-GACACGGATCTATACCAACGCC-3′; reverse: 5′-CCGTGA AGTTGTTCTCGGCGAA-3′.

The primers for the interleukin 1β (IL-1β) gene were as follows: forward: 5′-CCACAGACCTTCCAGGAGAATG-3′; reverse: 5′-GTGCAGTTCAGTGATCGTACAGG-3′.

The primers for the IL-6 gene were as follows: forward: 5′-AGACAGCCACTCACCTCTTCAG-3′; reverse: 5′-TTCTG CCAGTGCCTCTTTGCTG-3′.

The primers for the HOXA7 gene were as follows: forward: 5′-GCTGAGGCCAATTTCCGCATCT-3′; reverse: 5′-GTAGC GGTTGAAGTGGAACTCC-3′.

The primers for the HOXB4 gene were as follows: forward: 5′-CTGGATGCGCAAAGTTCACGTG-3′; reverse: 5′-CGTGT CAGGTAGCGGTTGTAGT-3′.

The primers for the GAPDH gene were as follows: forward: 5′-GTCTCCTCTGACTTCAACAGCG-3′; reverse: 5′-ACCAC CCTGTTGCTGTAGCCAA-3′.

### Protein Extraction and Western Blotting

Total proteins were extracted using whole-cell lysis buffer (Beyotime Biotechnology, Beijing, China). Twenty micrograms of protein from each sample was loaded into lanes and electrophoresed using 10% sodium dodecyl sulfate–polyacrylamide gel electrophoresis followed by transfer to a polyvinylidene difluoride (PVDF) membrane (0.45 μm; Millipore, Burlington, MA, United States). After blocking with 5% skimmed milk, the PVDF membranes were incubated with the primary antibody overnight at 4°C. Then, the PVDF membranes were incubated with the secondary antibodies at 25°C for 1 h. Protein expression was visualized with a chemiluminescence ECL reagent (Tanon, Woburn, MA, United States).

### Cell Proliferation Assay

The effects of drugs on cell proliferation were determined by the CCK8 Cell Proliferation Assay (Dojindo) according to the manufacturer’s instructions. Cells were plated in 96-well plates (three replicates per condition at 1 × 10^4^ cells per well) with different drugs and incubated at 37°C in 5% CO_2_. Then, 10 μL of CCK8 reagent was added directly to the wells, incubating for 2 h and recording the absorbance at 450 nm on an EnVision Plate Reader (Perkin Elmer, Waltham, MA, United States) at 0, 48, after drug administration. Data analysis was performed using Prism version 7.00 (GraphPad software). Unpaired Student *t* test was applied as the statistical method.

### Flow Cytometry of Cell Differentiation

The cells to be tested were taken and washed twice with phosphate-buffered saline (PBS). Then, these were resuspended in 500 μL PBS (the number of cells is controlled between 10^5^ and 10^6^); 2 μL CD11b antibody (APC channel) and 4 μL CD14 antibody (fluorescein isothiocyanate channel) were added to each tube, and the cells were incubated for 15 min at room temperature in the dark. After that, they were tested on the machine. FACS data were analyzed using FlowJo software.

### Statistical Analysis

R language (version 3.5.2) and GraphPad Prism 7 were mainly used for statistical analysis and figure drawing. Kaplan–Meier survival analysis and Cox regression analysis were used to indicate prognostic values. χ^2^ test was used for showing the difference of clinical features between two groups. Pearson correlation analysis was performed to test the correlation of two variants. Two-tailed *t* test was performed to calculate the quantitative difference between two groups. Volcano plots, forest plots, and survival curves were made by GraphPad Prism 7. Statistical significance was defined as *P* < 0.05.

## Results

### HOXB5 Is Upregulated and Acts as a Prognostic Marker in AML

**FIGURE 1:**
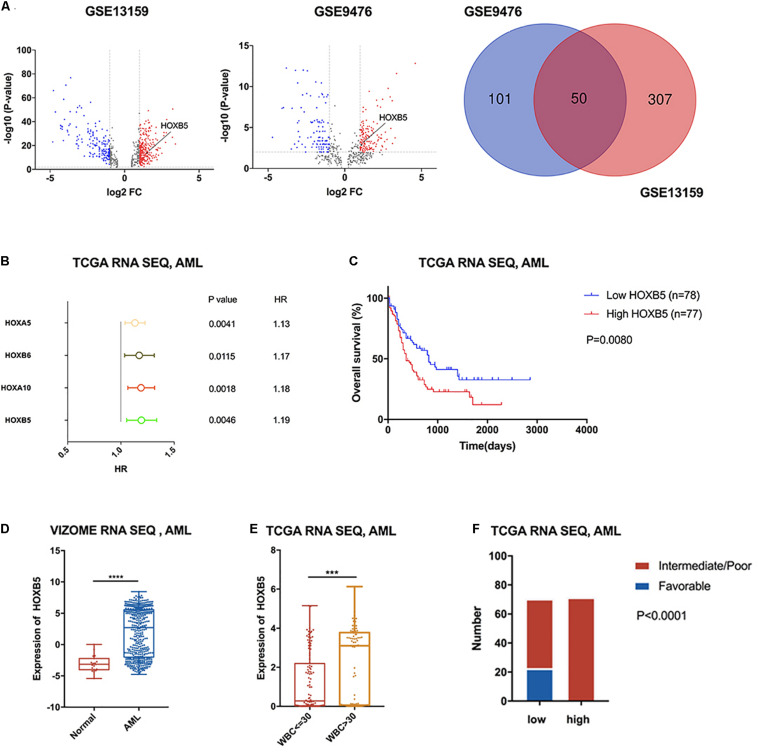
HOXB5 was up-regulated in AML with significant prognostic value and correlated with clinical characters of AML. **(A)** Volcano plots and a Venn plot showing DEGs between AML patients and healthy volunteers (GSE13159 RNA-seq, AML n1 = 542, normal n2 = 74; GSE9476 RNA-seq, AML n1 = 26, normal n2 = 10). **(B)** A forest plot showing Cox regression analyses of HOX genes included in the 50 overlapping genes using TCGA RNA-seq database. **(C)** Survival analysis revealed HOXB5 expressed prognostic value in AML (with log-rank test). **(D)** HOXB5 expression difference between AML patients and healthy volunteers (VIZOME, normal *n* = 19, AML *n* = 440; with *t* test). **(E)** The expression difference of HOXB5 between the groups above or below the set point (30 × 10^9^/L) of WBC count (with *t* test). **(F)** χ^2^ test showing the relationship between HOXB5 expression and cytogenetic risk in AML of TCGA RNA sequencing database. ***P < 0.001, ****P < 0.0001.

To identify dysregulated genes in AML, we performed differential gene expression analysis of samples from AML patients and healthy volunteers by employing the GSE13159 and GSE9476 datasets (Figure 1A). We found 50 overlapping differentially expressed genes (DEGs; *P* < 0.01, log_2_FC > 1) in both databases (Figure 1A and Supplementary Table 1). Among the 50 DEGs, four were members of the HOX family (HOXB5, HOXA5, HOXA10, and HOXB6). To test the prognostic value of HOX genes in AML patients, univariate Cox regression analysis of TCGA RNA sequencing data was performed (Supplementary Table 2). The results showed that high expression levels of most HOXA and HOXB family members indicated poor prognosis in AML. The four upregulated HOX genes were identified as significant by Cox regression analysis (Figure 1B). Next, we estimated the OS of AML patients stratified according to the expression of the four HOX genes with the log-rank test by employing the TCGA and VIZOME (Tyner et al., 2018) databases. HOXB5 was the only gene with prognostic value in all AML patients according to both Cox regression analysis and log-rank analysis (Figure 1C and Supplementary Figures 1,2A). In addition, HOXB5 expression levels could further stratify the AML patients with a normal karyotype or in different ELN risk groups (Supplementary Figures 2B–F).

To further investigate the clinical significance of HOXB5 in AML patients, we analyzed HOXB5 gene expression in different subgroups. Consistently, we found that the expression of HOXB5 in AML patients was increased significantly compared with that in healthy persons (Figure 1D). Furthermore, HOXB5 expression was positively associated with leukocytosis [white blood cell (WBC) count > 30 × 10^9^/L] and hyperleukocytosis (WBC count > 100 × 10^9^/L), which were biomarkers for inferior prognosis (Liu et al., 2020; Figure 1E and Supplementary Figures 3A,B). We then explored the connection between HOXB5 expression and cytogenetic risk and found that the patients in the favorable-risk group had lower HOXB5 expression than those in the intermediate-/poor-risk group (Figure 1F and Supplementary Figure 3C). These data indicate that high expression of the HOXB5 gene was a useful biomarker to predict inferior prognosis in AML patients.

### AML Patients With High HOXB5 Expression Have Distinct Somatic Mutation Patterns

**FIGURE 2:**
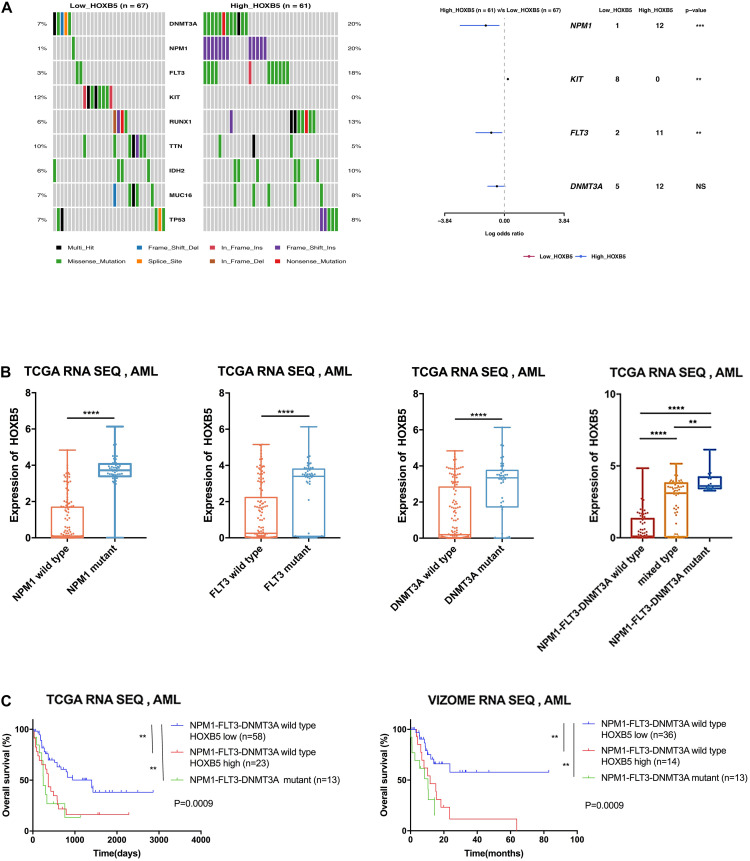
The correlation between somatic mutation manifestation and HOXB5 expression. **(A)** An oncoplot and a forest plot showing differential mutant genes between low and high HOXB5 expression groups using TCGA database. **(B)** The difference of HOXB5 expression between mutant and wild type in TCGA database (NPM1 wild type *n* = 128, NPM1 mutant *n* = 48; FLT3 wild type *n* = 127, FLT3 mutant *n* = 49; DNMT3A wild type *n* = 133, DNMT3A mutant *n* = 43; NPM1-FLT3-DNMT3A wild type *n* = 89; mixed type *n* = 73; NPM1-FLT3-DNMT3A mutant *n* = 14; with *t* test). **(C)** Survival curves showing HOXB5 expression correlated with significant prognosis in patients with NPM1-FLT3-DNMT3A wild type (with log-rank test). NS, no significance. **P < 0.01, *** *P* < 0.001, and ****P < 0.0001.

Previous investigations have demonstrated that several HOX genes are probably related to specific gene mutations, such as NPM1, DNMT3A, or MLL rearrangement (Armstrong et al., 2002; Falini et al., 2005; Yang et al., 2015). To determine the correlation between HOXB5 expression and somatic mutations, we analyzed somatic mutations and clinical information from the TCGA AML dataset. DNMT3A, FLT3, NPM1, and RUNX1 were the top four recurrent mutant genes in AML (Supplementary Figure 4A). We compared the differences between the somatic mutation frequencies of the low and high HOXB5 expression groups. The results showed that DNMT3A, FLT3, and NPM1 had higher mutation frequencies in the high HOXB5 expression group (Figure 2A). Consistently, the HOXB5 gene expression levels in AML patients with NPM1, FLT3, or DNMT3A mutations were higher than those in AML patients without gene mutations (Figure 2B and Supplementary Figure 4B). The AML patients with FLT3, NPM1, and DNMT3A (FND) triple mutations exhibited higher HOXB5 expression levels than AML patients with one or two gene mutations, whereas triple-negative AML patients (without any FLT3, NPM1, or DNMT3A mutations) showed the lowest HOXB5 expression levels (Figure 2B and Supplementary Figure 4B). These findings revealed that HOXB5 was closely related to NPM1, FLT3, and DNMT3A mutation status and could act as a biomarker of FND triple-mutant AML. Accumulated evidence has shown that FND triple-mutant AML represents a unique AML subset based on integrative genomic analysis and has an adverse prognosis (Ley et al., 2013; Papaemmanuil et al., 2016b). Intriguingly, we found that FND triple-mutant AML patients had poor prognosis, and high HOXB5 expression could further stratify FND triple-negative AML patients. FND triple-negative AML patients with high HOXB5 expression had a very poor prognosis similar to that of triple-mutant patients (Figure 2C). These data indicate that HOXB5 might play an important role in the pathogenesis of AML, and the underlying mechanism needs further investigation.

### HOXB5 Plays a Role in Myeloid Cell Differentiation Through the TNF/NF-κB Pathway and Is Correlated With the Leukemia Stem Cell Signature

**FIGURE 3:**
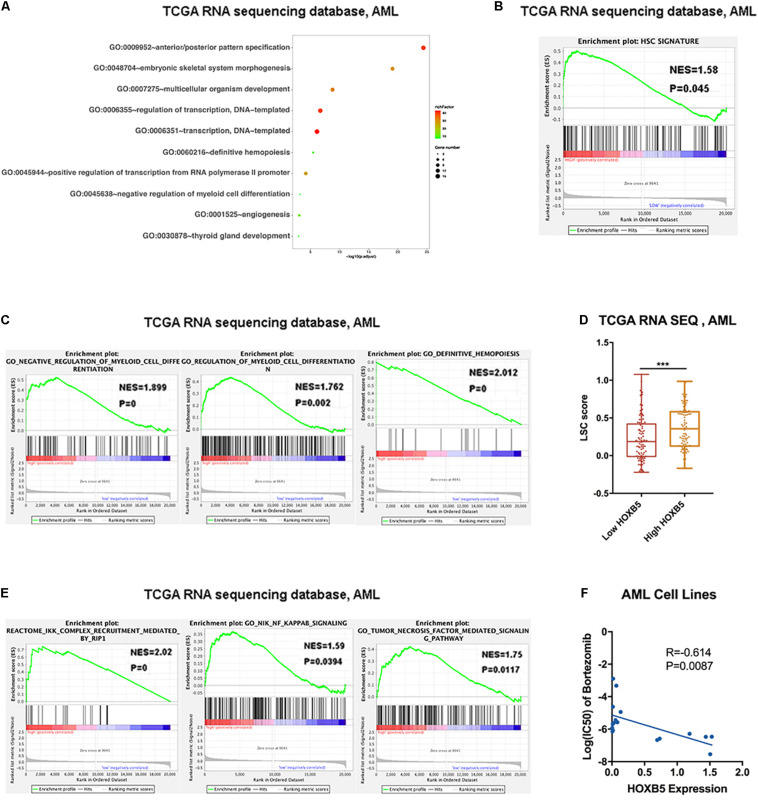
Downstream function analysis. **(A)** GO analysis showing the top 10 functions associated with HOXB5 positive-correlated genes in AML. **(B)** The result of GSEA verified HOXB5 acted in the HSC signature. **(C)** The results of GSEA verified HOXB5 acted in regulation of myeloid cells differentiation. **(D)** The difference of LSC score classified by HOXB5 expression. **(E)** The results of GSEA verified HOXB5 acted in the TNF/NF-κB pathway. **(F)** The correlation between HOXB5 expression and the IC50 of bortezomib in AML cell lines (with Pearson correlation analysis). ***P < 0.001.

First, we listed genes positively correlated with HOXB5 (*R* ≥ 0.5) in Supplementary Tables 3,4. GO analysis showed that HOXB5 was mainly related to definitive hematopoiesis and negatively associated with the regulation of myeloid cell differentiation (Figure 3A and Supplementary Figure 5A). GSEA indicated the association between HOXB5 expression and the hematopoietic stem cell (HSC) signature (Eppert et al., 2011), showing that high HOXB5 expression was related to upregulation of HSC-related genes (Figure 3B and Supplementary Figure 5B). Furthermore, higher HOXB5 expression was negatively associated with the myeloid cell differentiation signature (Figure 3C and Supplementary Figure 5C). Interestingly, the group with increased HOXB5 gene expression had a higher leukemia stem cell (LSC) score reported by a previous study (Ng et al., 2016), which indicated that HOXB5 might be important for LSC functions (Figure 3D and Supplementary Figure 5D). To further explore how HOXB5 functions in AML, we performed GSEA according to the signaling pathways. The results showed that HOXB5 was involved in the TNF/NF-κB pathway, which might subsequently regulate myeloid differentiation (Figure 3E and Supplementary Figure 5E). We then used the Cancer Cell Line Encyclopedia and Genomics of Drug Sensitivity in Cancer databases to analyze the correlation between HOXB5 gene expression and bortezomib, the clinically approved agent for multiple myeloma with activity to inhibit the NF-κB pathway (Shahshahan et al., 2011). Consistently, AML cell lines with higher HOXB5 expression levels were more sensitive to bortezomib than those with lower expression (Figure 3F).

**FIGURE 4:**
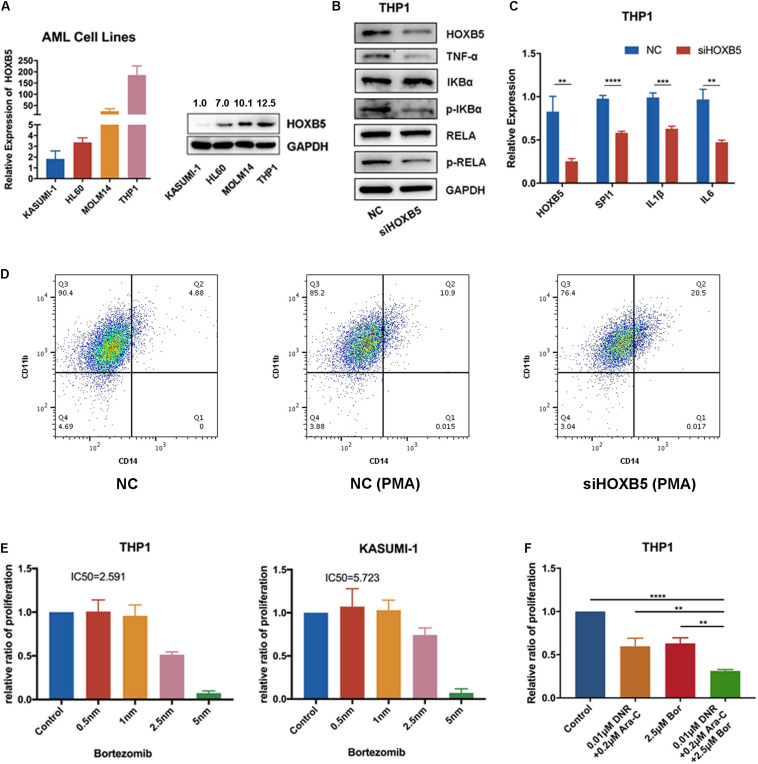
HOXB5 participates in myeloid cells differentiation by regulating TNF/NF-κB pathway. **(A)** HOXB5 expression differences in four AML cell lines. **(B)** The expression changes of TNF/NF-κB pathway related genes after HOXB5 knockdown confirmed by Western blot in THP1. **(C)** The expression changes of NF-κB–targeted genes after HOXB5 knockdown confirmed by RT-qPCR in THP1. **(D)** The cell differentiation changes of THP1 after PMA treatment between NC and siHOXB5 group. **(E)** The sensitivities of bortezomib in THP1 and KASUMI-1 (48 h). **(F)** The synergism effect of bortezomib and chemotherapy agents (daunomycin and cytarabine) in THP1 cells (48 h). **P < 0.01, ***P < 0.001, and ****P < 0.0001.

To verify our bioinformatics analysis results, we explored HOXB5 expression among four AML cell lines at both the RNA and protein levels and found that THP1 cells (with TP53 and NRAS mutations; without NPM1, FLT3, and DNMT3A mutations) had the highest expression level (Figure 4A). Then, we transfected si-HOXB5 and si-NC into THP1 cells, resulting in knockdown of HOXB5. We verified the transfection efficiency through reverse transcription (RT)–PCR and Western blot (Supplementary Figure 5F). To test the role of HOXB5 in the TNF/NF-κB pathway, key markers of this pathway were analyzed. We found that the expression of TNF-α, phosphorylated RELA, and phosphorylated IKBα was significantly downregulated after knockdown of HOXB5 (Figure 4B and Supplementary Figure 5G). To further explore the effect of HOXB5 on the NF-κB pathway, we detected several NF-κB–targeted genes, including SPI1, IL-1β, and IL-6, at the RNA level. We found that the expression of these genes decreased after HOXB5 knockdown (Figure 4C). Then, to verify the role of HOXB5 in myeloid cell differentiation, we detected CD11b and CD14 expression by flow cytometry and found that HOXB5 knockdown could promote the differentiation of THP1 cells upon PMA treatment (Figure 4D).

These findings suggest that HOXB5 may play roles in the maintenance of the LSC function and induction of myeloid cell differentiation through the TNF/NF-κB pathway.

To explore the clinical significance of HOXB5, we treated THP1 and KASUMI-1 cells with the NF-κB pathway inhibitor bortezomib and found that THP1 cells (with higher HOXB5 expression) were more sensitive to bortezomib than KASUMI-1 cells (with lower HOXB5 expression) (Figure 4E). Additionally, the combination of bortezomib and chemotherapy agents (daunomycin and cytarabine) showed enhanced effects on THP1 cells (Figure 4F).

### HOXB5-Correlated HOX Family Members Function Together With HOXB5 in the Pathogenesis of AML

**FIGURE 5:**
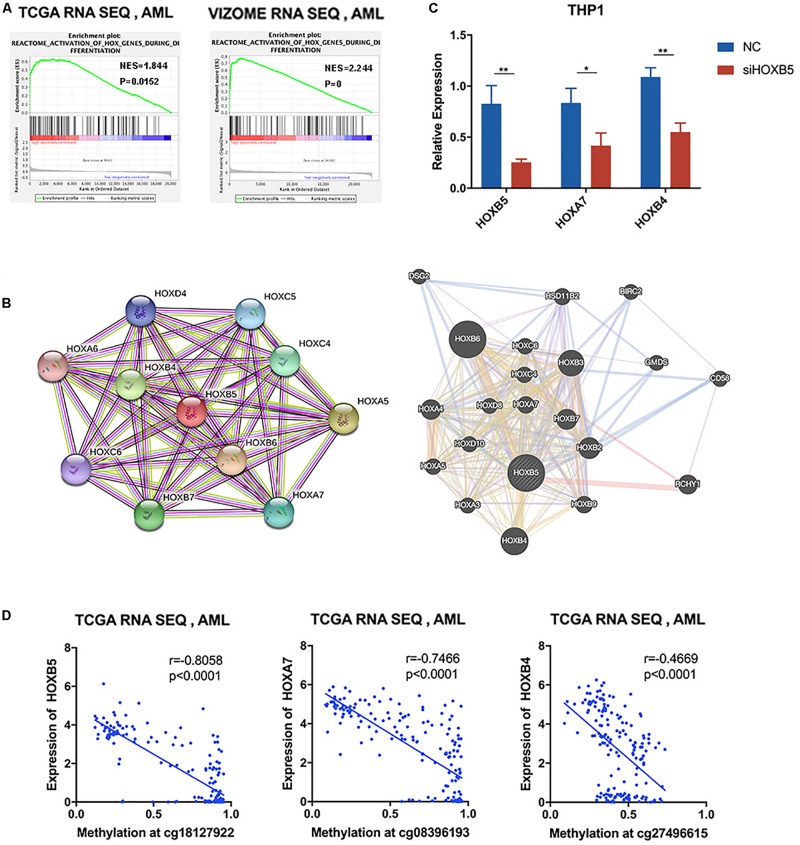
HOXB5-related homeobox genes functioned together with HOXB5 in AML. **(A)** GSEA analyses verifying the function of HOXB5 in activation of HOX genes during differentiation. **(B)** Protein–protein interaction of HOXB5 using STRING and GeneMANIA. **(C)** The expression changes of HOXB5 and its related HOX genes (HOXA7 and HOXB4) after knockdown of HOXB5 (with *t* test). **(D)** The correlation between HOXB5, HOXA7, and HOXB4 expression and their methylation level at locus cg18127922, cg08396193, and cg27496615, respectively, with methylation data of TCGA (with Pearson correlation analysis). *P < 0.05, **P < 0.01.

In the list of genes closely correlated with HOXB5, we found many other HOX family members. Therefore, the correlated members enriched in the functions of myeloid cell differentiation and definitive hematopoiesis were analyzed, and the results verified the concordance among the selected HOX genes (Supplementary Figure 6A). GSEA indicated that HOXB5 was associated with the activation of HOX genes during differentiation in AML (Figure 5A). Moreover, the protein–protein interaction network of HOXB5 with the STRING and GeneMANIA online tools demonstrated the correlation between HOXB5 and some other HOX members at the protein level (Figure 5B). In addition, we found that two related HOX genes (HOXA7 and HOXB4) were both downregulated after knockdown of HOXB5 (Figure 5C). These findings suggest that HOXB5 and its related HOX genes may function together in the pathogenesis of AML and that HOXB5 may be the center of this regulatory system.

As HOX genes are clustered in an ordered chromosomal arrangement and recent studies indicated that HOX genes could be regulated by DNA methylation (Paço et al., 2020), we further analyzed the correlation between DNA methylation status and gene expression levels of HOX genes in the AML TCGA database. The results showed that HOXB5 gene expression, as well as the expression of its related genes HOXA7 and HOXB4, was negatively correlated with its DNA methylation status (Figure 5D). In addition, we did not find any somatic mutations in the HOXB5, HOXA7, and HOXB4 genes of AML patients (Supplementary Table 5). Additionally, the CNV alterations of these genes did not have a relationship with gene expression (Supplementary Table 6 and Supplementary Figure 6B). Therefore, the results indicate that these HOX gene expression levels were not regulated by their CNV or mutation status.

These findings suggest that DNA methylation may be a common upstream mechanism regulating the network of HOX family genes, including HOXB5 and its related genes.

## Discussion

Currently, chromosomal variations and gene mutations are considered the main factors in the pathogenesis of AML and are combined to assess the cytogenetic risk for prognostic prediction and treatment selection (Alharbi et al., 2013; Meyer and Levine, 2014; Döhner et al., 2017; Nagy et al., 2019). However, nearly 50% of AML patients harbor a normal karyotype, and some of them even lack common somatic mutations (Ibáñez et al., 2020). Thus, it is important to explore new potential factors with prognostic significance and/or roles in leukomogenesis. In this study, we tried to integrate transcriptomic data with clinical features in AML patients and found that HOXB5 might be a suitable biomarker.

Previous studies have demonstrated that several HOX family members affect the poor prognosis and aggressiveness of AML through transcriptional regulation (Andreeff et al., 2008; Zangenberg et al., 2009; Collins and Hess, 2016). However, the roles of HOX genes in AML have not been comprehensively studied. Here, by digging deeply into transcriptomic data, we identified HOXB5 as the only significant gene highly expressed in AML patients, which had prognostic value based on clinical features and survival analysis. These data support that HOXB5 could be a useful biomarker for AML prognostic prediction. Interestingly, we found that elevated HOXB5 expression in AML was associated with definitive hematopoiesis and myeloid cell differentiation, as well as the LSC signature. Consistently, two previous studies revealed that HOXB5 was an essential gene for maintaining the long-term self-renewal capacity of HSCs (Chen et al., 2016; Sakamaki et al., 2021). Therefore, we rationally speculated that AML cells with high HOXB5 expression might have characteristics of LSCs, conferring drug resistance and inferior prognosis to patients. HOXB5, as a transcription factor, is an undruggable target, so targeting the HOXB5 pathway might be an alternative strategy. We found that HOXB5 might regulate the TNF/NF-κB pathway, indicating potential targets for AML treatment. The relationship between HOX genes and NF-κB is not clear now. As HOXB5 is a known transcription factor, we speculate that HOXB5 possibly regulates the expression of key genes in the TNF/NF-κB pathway, the molecular mechanism of which needs further exploration. Indeed, it was shown that HOXB5 expression was positively correlated with the sensitivity of AML cells to bortezomib, an NF-κB pathway inhibitor in our data (Shahshahan et al., 2011). In recent years, increased expression and activation of NF-κB signaling components have been observed in AML cells (Oeckinghaus et al., 2011; Gasparini et al., 2014; Darwish et al., 2019). Therefore, we proposed that HOXB5 plays an essential role in the pathogenesis of AML by regulating myeloid cell differentiation and maintaining LSC function. The TNF/NF-κB pathway is one of the key points and could be a potential therapeutic target for AML with high HOXB5 expression. We found that NF-κB pathway inhibitors could synergize with chemotherapy agents (daunomycin and cytarabine) to inhibit the proliferation of THP1 cells. Therefore, combining NF-κB pathway inhibitors with current treatments might improve the therapeutic efficacy and survival of AML patients, especially those with high HOXB5 expression.

Genetic mutations have significantly contributed to the optimization of not only classification but also risk stratification, residual disease monitoring, and even the development of targeted therapeutics (Eisfeld et al., 2020). FLT3-ITD, NPM1, and DNMT3A mutations frequently occur in AML patients, and the combination of these three gene mutations (FND triple-mutant AML) was reported to confer inferior survival and a higher risk of relapse (Loghavi et al., 2014; Papaemmanuil et al., 2016a,b). Recent studies have indicated that FND triple-mutant AML might be a subtype of AML with unique characteristics at the mRNA, miRNA, and epigenetic levels (Ley et al., 2013). High expression of several HOX genes was associated with NPM1 or DNMT3A mutations (Falini et al., 2005; Mullighan et al., 2007; Yang et al., 2015). Here, we found that NPM1, FLT3, and DNMT3A mutations were more likely to occur in AML patients with higher HOXB5 expression. Compared with FND wild-type or mixed-mutant groups (carrying one or two genetic mutations of NPM1, FLT3, or DNMT3A), HOXB5 expression was highest in the FND triple-mutant group. This result indicates that HOXB5 may be a common downstream gene and can be a potential biomarker for FND triple-mutant AML. Additionally, intermediate/high risk–defined cytogenetic/genetic abnormalities were more likely to occur in AML patients with higher HOXB5 expression. These data suggest that the expression level of HOXB5 was correlated with certain genetic background in AML; therefore, HOXB5 expression was not an independent prognostic factor. Interestingly, we found that HOXB5 could also further stratify FND wild-type patients, and those with higher HOXB5 expression experienced a poor prognosis similar to that of triple-mutant patients. However, the upstream molecular mechanism of this population of AML patients remains unclear and needs further investigation.

As transcription factors, some HOX genes have been found to activate their own expression or to cross-regulate their family members or their cofactors (Alharbi et al., 2013). We found that HOXB5 was closely correlated with the expression of some other HOX family members enriched in the same AML-related function. While HOX genes have been reported to be regulated by the MLL protein and the Cdx protein (Armstrong et al., 2002; Drabkin et al., 2002; Eklund, 2011), we found that the expression levels of HOXB5 and two of its related members (HOXA7 and HOXB4) were negatively associated with their DNA methylation levels. These findings imply that the regulation of HOX genes was complicated, and DNA methylation may be one of the pathways.

Taken together, HOXB5 was indicated to be upregulated in patients with AML, which was associated with the progression of AML and inferior prognosis, even in different subgroups. The AML patients with higher HOXB5 expression levels had unique genetic mutation patterns, such as concurrent FLT3-ITD, NPM1, and DNMT3A mutation patterns. In AML patients, higher expression of HOXB5 was closely related to myeloid differentiation and LSC characteristic gene signatures, probably through the TNF/NF-κB pathway. DNA methylation of clustering HOX genes might be one of the regulatory mechanisms that need further investigation. These results suggest that HOXB5 may serve as a novel biomarker for predicting the clinical outcome of patients with AML. However, further investigations are required to determine the biological characteristics of HOXB5 and their potential mechanism involved in leukemogenesis.

## Data Availability Statement

The original contributions presented in the study are included in the article/Supplementary Material, further inquiries can be directed to the corresponding author/s.

## Author Contributions

MC, YQ, PY, and XY contributed to the study conceptualization. MC contributed to the data curation and took charge of the methodology and the validation. XY contributed to the funding acquisition and supervised the study and took charge of the review and editing. PY contributed to the project administration. MC and YQ contributed to writing the original draft. All authors contributed to the article and approved the submitted version.

## Conflict of Interest

The authors declare that the research was conducted in the absence of any commercial or financial relationships that could be construed as a potential conflict of interest.

## Publisher’s Note

All claims expressed in this article are solely those of the authors and do not necessarily represent those of their affiliated organizations, or those of the publisher, the editors and the reviewers. Any product that may be evaluated in this article, or claim that may be made by its manufacturer, is not guaranteed or endorsed by the publisher.

## Footnotes

1
https://www.ncbi.nlm.nih.gov/geo/query/acc.cgi?acc=GSE13159

2
https://www.ncbi.nlm.nih.gov/geo/query/acc.cgi?acc=GSE9476

3
https://david.ncifcrf.gov/tools.jsp

4
http://www.broadinstitute.org/gsea/index.jsp
