# Supplementary material for: The Prognostic Value and Function of HOXB5 in Acute Myeloid Leukemia
Source: Front Genet. 2021 Aug 5;12:678368. doi: 10.3389/fgene.2021.678368 (PMC8376581; doi:10.3389/fgene.2021.678368)
Supplement: Supplementary file 1 [file Data_Sheet_1.DOCX]

**Supplementary Figure Legends**

**Supplementary Figure 1. Survival curves of the other three elevated HOX genes.**

(**A**) Survival analysis revealed HOXA5 expressed prognostic value in TCGA database, but not in VIZOME database (with log-rank test).

(**B**) Survival analysis revealed HOXA10 expressed prognostic value in TCGA database, but not in VIZOME database (with log-rank test).

(**C**) Survival analysis revealed HOXB6 expressed prognostic value in TCGA database, but not in VIZOME database (with log-rank test).

**Supplementary Figure 2. HOXB5 further predicted the prognosis of patients identified by traditional prognostic markers.**

(**A**) Survival analysis revealed HOXB5 expressed prognostic value in VIZOME database (with log-rank test).

(**B**) Survival curves showing HOXB5 expression correlated with significant prognosis in patients with normal karyotype in TCGA database (with log-rank test).

(**C**) Survival curves showing HOXB5 expression correlated with significant prognosis in patients with normal karyotype in VIZOME database (with log-rank test).

(**D**) Survival curves showing HOXB5 expression correlated with significant prognosis in patients in intermediate risk group in TCGA database (with log-rank test).

(**E**) Survival curves showing HOXB5 expression correlated with significant prognosis in patients in intermediate risk group in VIZOME database (with log-rank test).

(**F**) Survival curves showing HOXB5 expression correlated with significant prognosis in patients in favorable risk group in VIZOME database (with log-rank test).

**Supplementary Figure 3. HOXB5 was correlated with clinical malignant characters.**

(**A**) The expression difference of HOXB5 between the groups above or below the set point (30*10^9^) of WBC in VIZOME database (with t test).

(**B**) The expression difference of HOXB5 between the groups above or below the set point (100*10^9^) of WBC in TCGA and VIZOME database (with t test).

(**C**) HOXB5 expression difference among favorable, intermediate and poor risk group classified by cytogenetic risk evaluation (with t test).

*, P<0.05; ***, P<0.001; ****, P<0.0001.

**Supplementary Figure 4. The correlation between somatic mutation manifestation and HOXB5 expression.**

(**A**) An oncoplot showing overall mutant genes manifestation using TCGA database.

(**B**) The difference of HOXB5 expression between mutant and wild type in VIZOME database (NPM1 wild type n=157, NPM1 mutant n=65; FLT3 wild type n=166, FLT3 mutant n=56; DNMT3A wild type n=67, DNMT3A mutant n=34; NPM1-FLT3-DNMT3A wild type n=46; mixed type n=45; NPM1-FLT3-DNMT3A mutant n=10; with t test).

ns, no significance; **, P<0.01; ****, P<0.0001.

**Supplementary Figure 5. Downstream function analysis.**

(**A**) GO analysis showing the top10 functions associated with HOXB5 positive-correlated genes in VIZOME database.

(**B**) The result of GSEA verified HOXB5 acted in the HSC signature in VIZOME database.

(**C**) The results of GSEA verified HOXB5 acted in regulation of myeloid cells differentiation in VIZOME database.

(**D**) The difference of the LSC score classified by HOXB5 expression in VIZOME and GSE13159 database (with t test).

(**E**) The results of GSEA verified HOXB5 acted in the TNF/NF-κB pathway in VIZOME database.

(**F**) The knockdown efficiency of HOXB5 expression confirmed by PCR and Western blot.

(**G**) The expression changes of TNF/NF- κB pathway related genes after HOXB5 knockdown confirmed by Western blot in THP1.

*, P<0.05; **, P<0.01; ***, P<0.001; ****, P<0.0001.

**Supplementary Figure 6. HOXB5-related homeobox genes.**

(**A**) Hotmaps showing the association of HOXB5 expression with age, gender, FAB subtype and other nine homeobox genes (TCGA RNA sequencing database, n=179; VIZOME RNA sequencing database, n=210; with Pearson correlation analysis).

(**B**) CNV status of HOXB5, HOXA7 and HOXB4 (with t test).

ns, no significance.
