# Supplementary figures and images for: The Prognostic Value and Function of HOXB5 in Acute Myeloid Leukemia
Source: Front Genet. 2021 Aug 5;12:678368. doi: 10.3389/fgene.2021.678368 (PMC8376581; doi:10.3389/fgene.2021.678368)

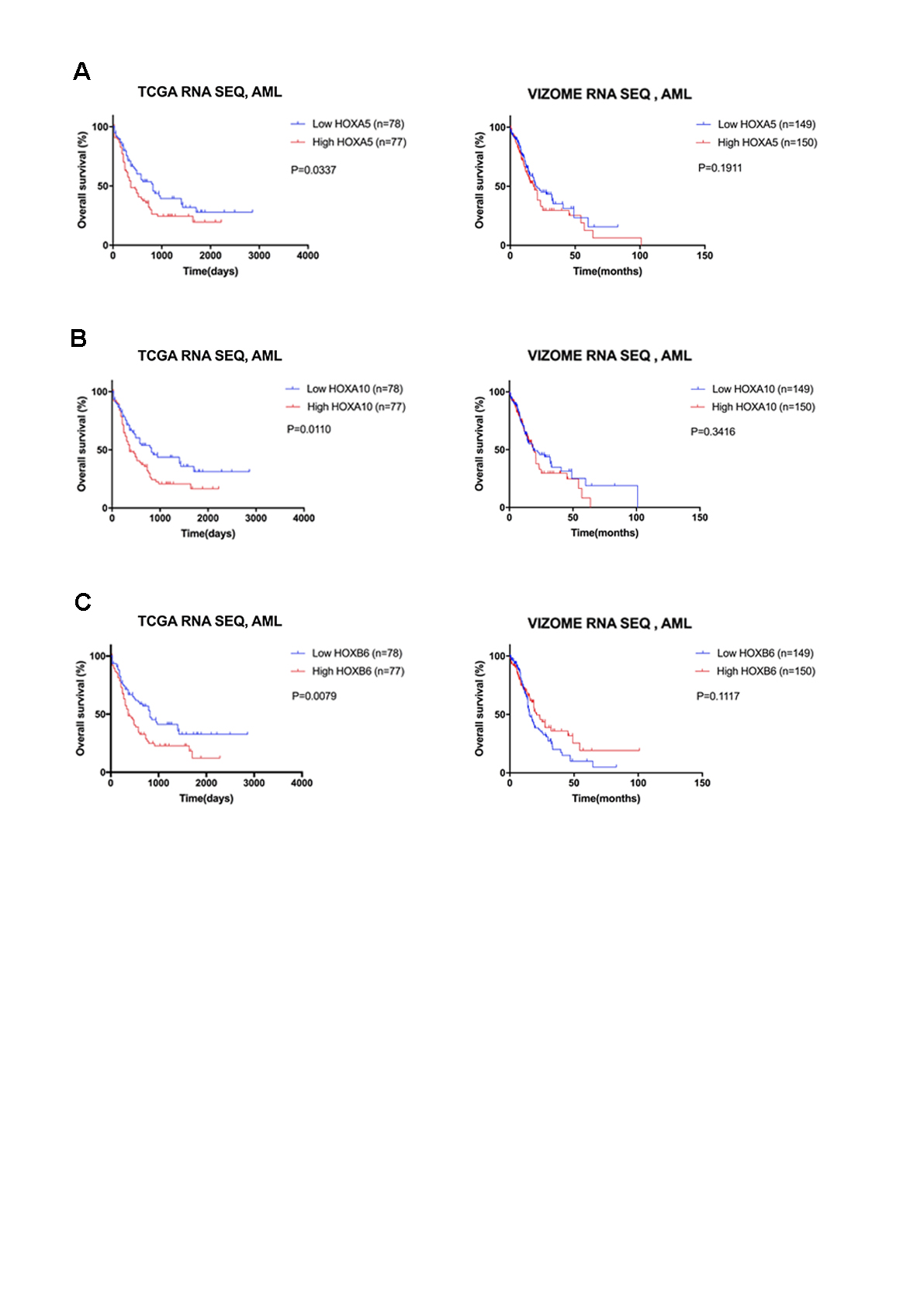

Supplement: Supplementary file 2 [file Image_1.JPEG]

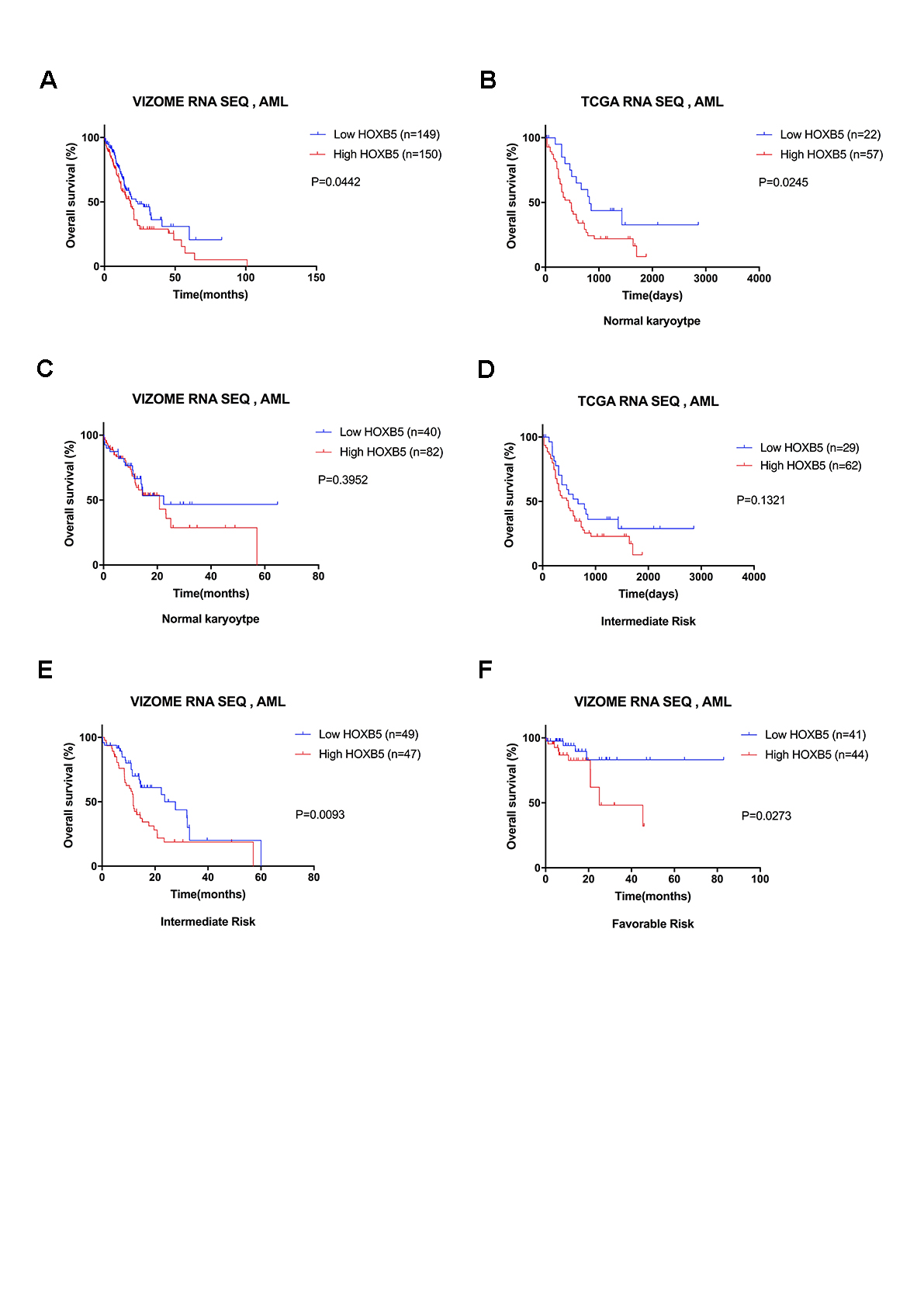

Supplement: Supplementary file 3 [file Image_2.JPEG]

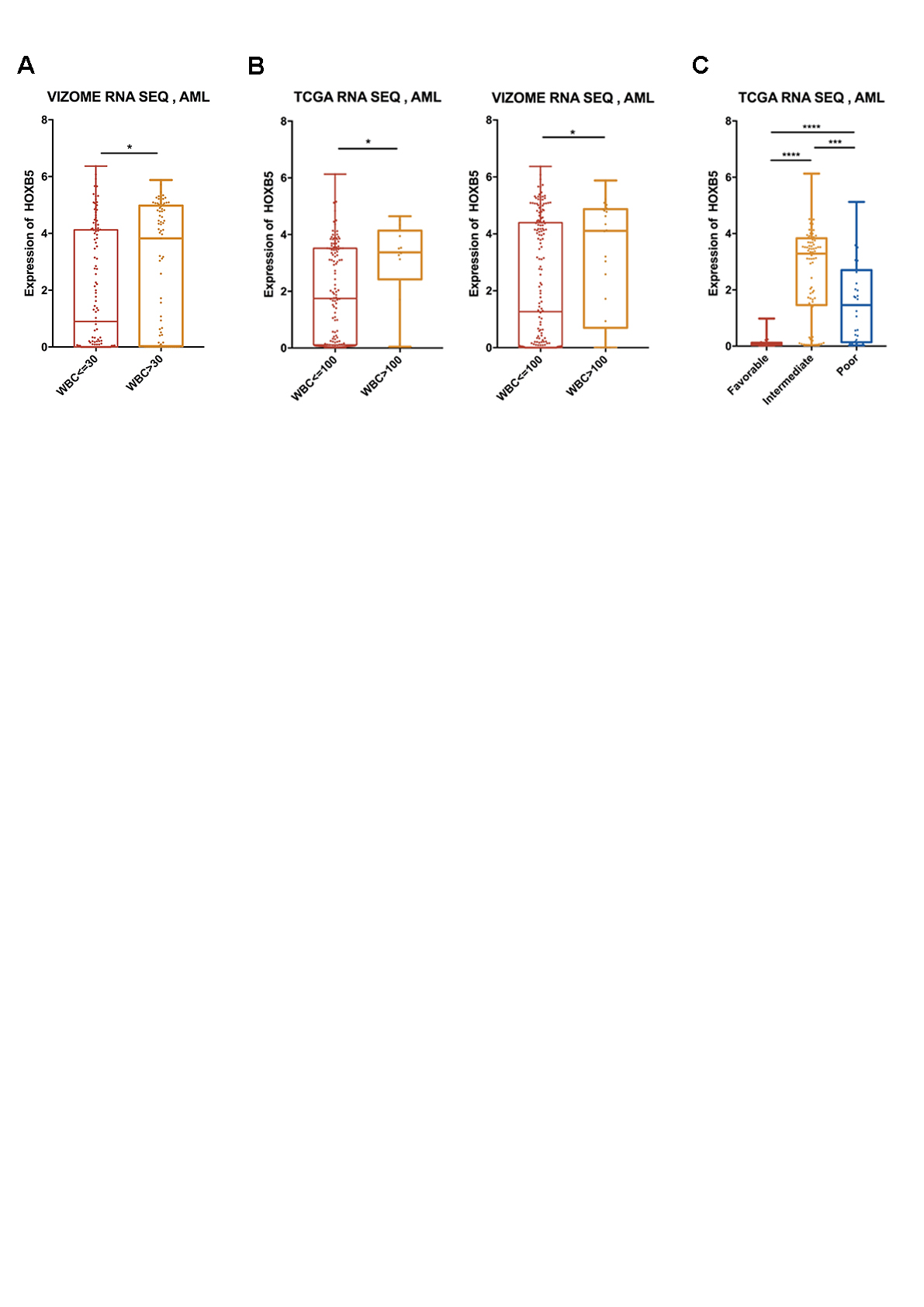

Supplement: Supplementary file 4 [file Image_3.JPEG]

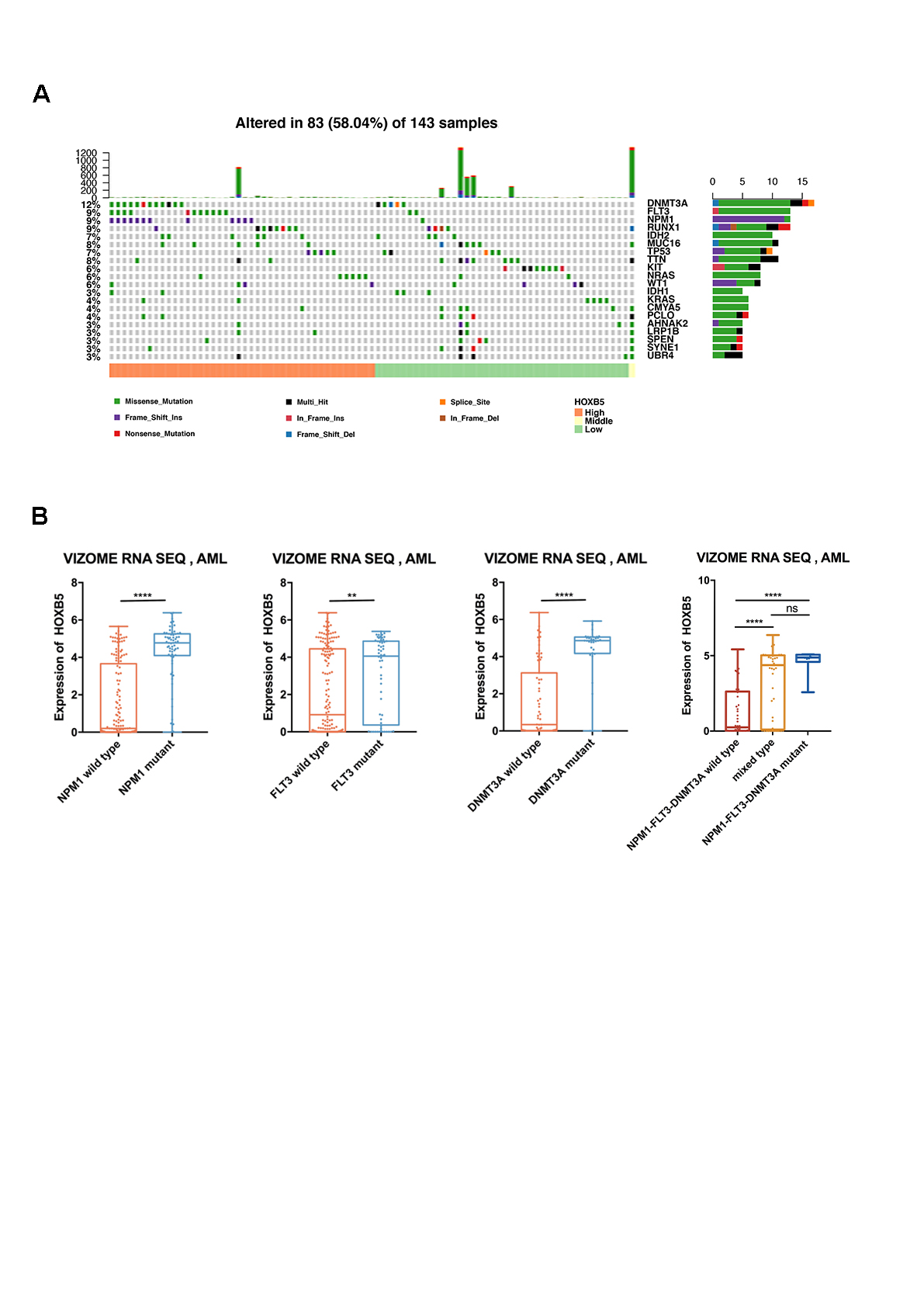

Supplement: Supplementary file 5 [file Image_4.JPEG]

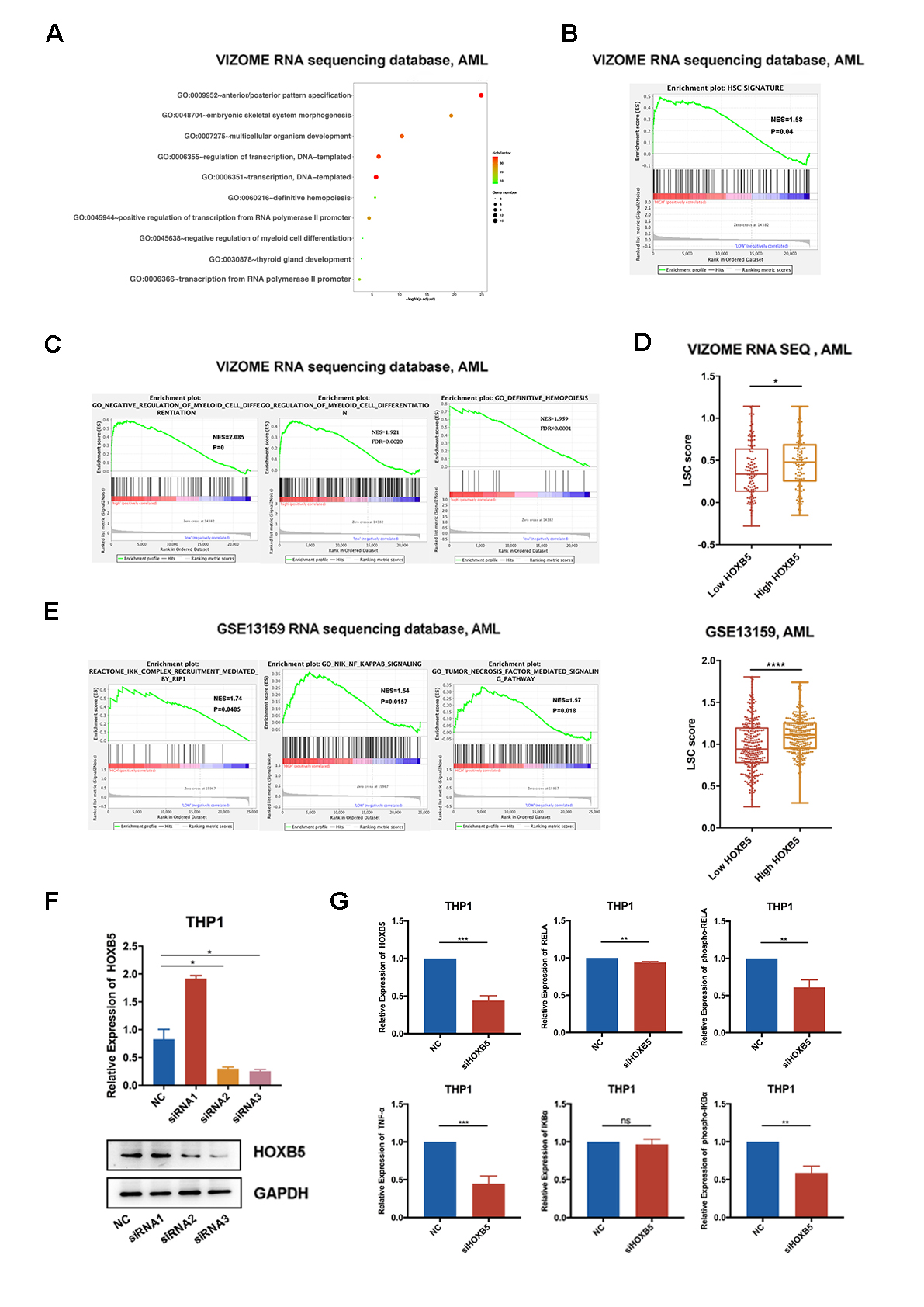

Supplement: Supplementary file 6 [file Image_5.JPEG]

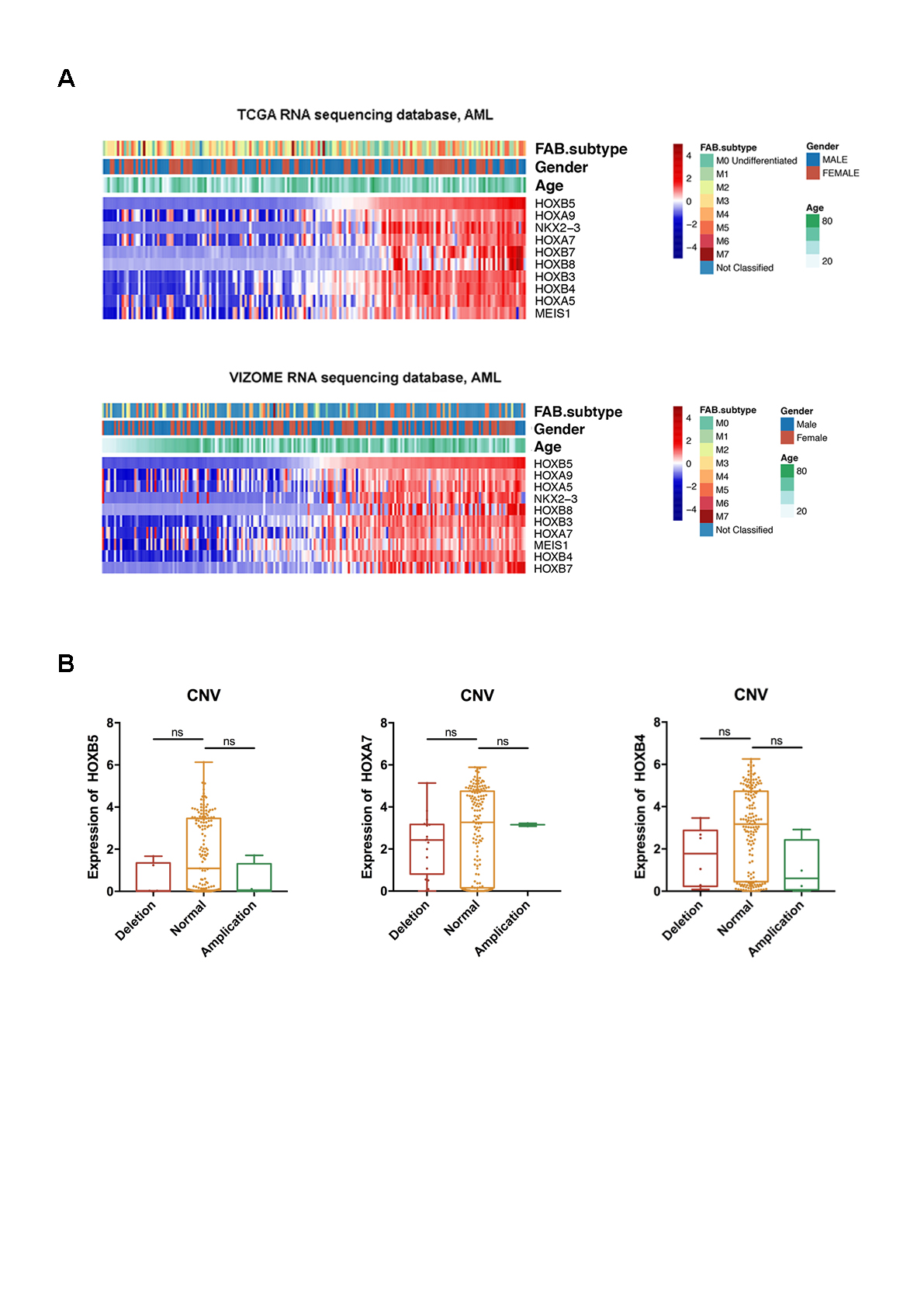

Supplement: Supplementary file 7 [file Image_6.JPEG]
