# Supplementary material for: The Prognostic Value and Function of HOXB5 in Acute Myeloid Leukemia
Source: Front Genet. 2021 Aug 5;12:678368. doi: 10.3389/fgene.2021.678368 (PMC8376581; doi:10.3389/fgene.2021.678368)
Supplement: Supplementary file 9 [file Table_2.PDF]

Gene	HR	P value
HOXA1	1.3226	0.0679
HOXA2	1.1750	0.0896
HOXA3	1.1646	0.0081
HOXA4	1.1470	0.0247
HOXA5	1.1292	0.0041
HOXA6	1.1376	0.0060
HOXA7	1.1786	0.0014
HOXA9	1.1594	0.0005
HOXA10	1.1842	0.0018
HOXA11	1.0545	0.5689
HOXA13	1.0369	0.8454
HOXB1	2.0777	0.2829
HOXB2	1.1361	0.0284
HOXB3	1.0960	0.0390
HOXB4	1.1193	0.0328
HOXB5	1.1867	0.0046
HOXB6	1.1660	0.0115
HOXB7	1.2190	0.0262
HOXB8	1.1619	0.0499
HOXB9	1.1605	0.0399
HOXB13	4.02E+11	0.0049
HOXC4	1.1761	0.4921
HOXC5	0.8169	0.6696
HOXC6	0.8826	0.6313
HOXC8	0.8471	0.8034
HOXC9	0.7379	0.4527
HOXC10	1.0782	0.7955
HOXC11	0.2113	0.3292
HOXC12	0.4587	0.4077
HOXC13	2.26E-06	0.3499
HOXD1	6.4008	0.7497
HOXD3	0.3583	0.7344
HOXD4	0.3171	0.4625
HOXD8	1.1762	0.8903
HOXD9	1.3363	0.8570
HOXD10	0.0011	0.3317
HOXD11	3.57E-05	0.3637
HOXD12	0.1928	0.3685
HOXD13	0.4182	0.4355
