# Supplementary material for: The Prognostic Value and Function of HOXB5 in Acute Myeloid Leukemia
Source: Front Genet. 2021 Aug 5;12:678368. doi: 10.3389/fgene.2021.678368 (PMC8376581; doi:10.3389/fgene.2021.678368)
Supplement: Supplementary file 10 [file Table_3.PDF]

HOXB5	FLJ42875
LOC441204	C22orf26
ABHD11	AIFM2
PBX3	SDSL
HOXA4	SLC38A10
HOXA3	WNT7B
HOXA2	EMR1
HOXA1	SLC25A28
HOXA10	NRG4
ARHGAP22	SPATA2L
PRDM16	HOXA6
LOC100271722	HOXB8
CPNE8	HOXB9
HOXA9	HOXB2
NKX2-3	HOXB3
RIPK3	HOXB6
C7orf50	HOXB4
HOXA7	LOC400931
PHKA2	UGGT2
HOXB7	HOXA5
C1QL1	MEIS1
C10orf140	PDGFD
