# Supplementary material for: The Prognostic Value and Function of HOXB5 in Acute Myeloid Leukemia
Source: Front Genet. 2021 Aug 5;12:678368. doi: 10.3389/fgene.2021.678368 (PMC8376581; doi:10.3389/fgene.2021.678368)
Supplement: Supplementary file 11 [file Table_4.PDF]

HOXB5	HOXB9	RP11-129J12.2
ADCY2	PDGFD	HOXB7
HOXA9	HOXB2	RP11-1055B8.4
UGGT2	EMR1	RP11-1055B8.6
HOXA1	LINC00982	RP1-170O19.21
HOXA2	C3orf80	NRG4
HOXA3	HOXB4	MIR10A
HOXA5	WNT7B	
HOXA6	FLJ27365	
HOXB6	HOXA4	
NKX2-3	OCLN	
HOXB8	RP11-129J12.1	
HOXB3	HOXB-AS1	
HOXA7	RP6-109B7.2	
C1QL1	LINC00899	
CPNE8	RP11-388P9.2	
SDSL	HOXB-AS3	
PRDM16	RP6-109B7.3	
MEIS1	IL12A-AS1	
MPP7	HOXA10-AS	
IGSF10	HOXA10	
PBX3	HOXA-AS3	
